# Correction: Archibong, A.E., *et al*. Effects of Benzo(a)pyrene on Intra-testicular Function in F-344 Rats

**DOI:** 10.3390/ijerph7031224

**Published:** 2010-03-22

**Authors:** Anthony E Archibong, Aramandla Ramesh, Mohammad S Niaz, Cynthia M Brooks, Shannon I Roberson, Donald D Lunstra

**Affiliations:** 1 Department of Obstetrics and Gynecology, Meharry Medical College, Nashville, TN 37208, USA; 2 Department of Biochemistry and Cancer Biology, Meharry Medical College, Nashville, TN 37208, USA; 3 U.S. Meat Animal Research Center, USDA ARS, Clay Center, NE 68933, USA

We found some errors in Figure 4 in our paper published in the *International journal of Environmental Research and Public Health* [1]. Figure 4 is corrected as follows: the values on the Y axis should be in ng/gm testis and not ug/gm testis. Secondly, it is indicated in the body of the manuscript that the ITT values are represented per gram of testis but in Figure 4 the values reported was not normalized per gram of testis weight. The values on Figure 4 are ITT per testis. So we divided these values by the testis weight to arrive at the values represented in the graph below.

**Figure 4.**
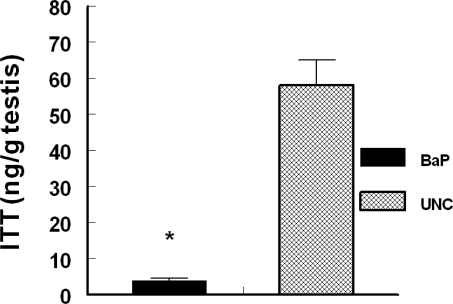
Effect of inhaled BaP on ITT concentrations in F-344 male rats exposed to 75 μg BaP/m^3^ for 60 days; *n* = 10 per treatment or control group. Results are expressed as mean + SE (UNC = unexposed control; BaP = BaP-inhaled rats. Asterisks indicate a significant difference from controls P < 0.05).

We apologize for any inconvenience caused to the readers.
